# Exerkines and brain rejuvenation

**DOI:** 10.1002/alz.083404

**Published:** 2025-01-03

**Authors:** Odette Leiter, Joshua Lowe, David Brici, Tara L Walker

**Affiliations:** ^1^ The University of Queensland, Brisbane, QLD Australia

## Abstract

Ageing changes the adult brain at the molecular, cellular and functional levels, driving regenerative decline, inflammation, cognitive impairments and susceptibility to dementia‐related neurodegenerative disorders, such as Alzheimer’s disease (AD). There is overwhelming evidence that regular physical exercise can counteract cognitive decline in both healthy ageing and in neurodegenerative conditions such as AD, with exerkines, the circulating humoral factors that are secreted into the blood stream in response to exercise, emerging as likely mediators of this response. However, the source and identity of these exerkines remain unclear. We have recently found that platelets, the small immune cells that primarily regulate haemostasis, are activated after acute periods of running and release factors, including the chemokine platelet factor 4 (PF4), that promote neurogenesis and cognitive rejuvenation.

This presentation will highlight the key role that platelet‐derived exerkines play in mediating cognitive rejuvenation and suggest that platelets represent a unique and underexplored therapeutic approach to counteract age‐related cognitive decline.

